# A Role for *SPARC* in the Moderation of Human Insulin Secretion

**DOI:** 10.1371/journal.pone.0068253

**Published:** 2013-06-28

**Authors:** Lorna W. Harries, Laura J. McCulloch, Janet E. Holley, Thomas J. Rawling, Hannah J. Welters, Katarina Kos

**Affiliations:** Institute of Biomedical and Clinical Sciences, Exeter Medical School, University of Exeter, Exeter, United Kingdom; Wageningen University, The Netherlands

## Abstract

**Aims/Hypothesis:**

We have previously shown the implication of the multifunctional protein SPARC (Secreted protein acidic and rich in cysteine)/osteonectin in insulin resistance but potential effects on beta-cell function have not been assessed. We therefore aimed to characterise the effect of SPARC on beta-cell function and features of diabetes.

**Methods:**

We measured *SPARC* expression by qRT-PCR in human primary pancreatic islets, adipose tissue, liver and muscle. We then examined the relation of *SPARC* with glucose stimulated insulin secretion (GSIS) in primary human islets and the effect of *SPARC* overexpression on GSIS in beta cell lines.

**Results:**

*SPARC* was expressed at measurable levels in human islets, adipose tissue, liver and skeletal muscle, and demonstrated reduced expression in primary islets from subjects with diabetes compared with controls (p< = 0.05). SPARC levels were positively correlated with GSIS in islets from control donors (p< = 0.01). Overexpression of *SPARC* in cultured beta-cells resulted in a 2.4-fold increase in insulin secretion in high glucose conditions (p< = 0.01).

**Conclusions:**

Our data suggest that levels of *SPARC* are reduced in islets from donors with diabetes and that it has a role in insulin secretion, an effect which appears independent of SPARC’s modulation of obesity-induced insulin resistance in adipose tissue.

## Introduction

The *SPARC* gene encodes a multifunctional matricellular protein which modulates the interaction between cells and the extra-cellular matrix (ECM) by the regulation of factors such as collagen and vitronectin [1]. SPARC has roles in many processes, including wound healing, inflammation, extracellular protease activity, angiogenesis, remodelling of cardiac muscle and modulation of growth factor signalling [2]. Alterations to SPARC levels or activity have been associated with several common human diseases such as osteoporosis [3], arthritis [4], [5], cancer [6], heart disease [7], [8], obesity [8], [9], and independent of obesity, type 2 diabetes [10], [11]. *SPARC* is expressed at relatively high levels in most tissues, but particularly in adipose tissue, where it inhibits adipogenesis [12], [13].

The influence of SPARC in insulin resistance and type 2 diabetes may arise from its role in the regulation of collagen assembly and ECM remodelling [14], [15]. *SPARC* is expressed and secreted from adipose tissue at higher levels in obese individuals than in lean individuals [9], furthers ECM fibrosis which in turn impairs adipocyte differentiation and adipose tissue expansion [10], [16]. Restriction of adipose tissue expansion in combination with surplus dietary lipid exposure leads to systemic hyperlipidaemia and ultimately triglyceride infiltration of other organs, as ectopic fat [12] which furthers insulin resistance. SPARC circulating levels are associated with HOMA-IR [9] and HbA1c [17] in obese individuals without diabetes and secretion from adipose tissue is induced by insulin. These data point to an important role for SPARC in insulin resistance. SPARC also has potential to act as a regulator of insulin secretion or in maintenance of beta-cell function. It is known to be involved in WNT signalling, which is important in control of cell-cell communication and cellular proliferation and differentiation [18], and also regulates several genes involved in beta-cell development or islet neogenesis [19], [20], [21] and is also known to regulate cell survival [22], [23]. These observations make SPARC an interesting candidate as a modifier of islet insulin secretory capacity, since both destruction and dedifferentiation of beta cells has been described in type 2 diabetes [24], [25].

The overall aim of this study is to provide evidence of SPARC’s role on beta-cell function in human physiology and study of primary human tissues. We aimed to assess the potential for *SPARC* to be involved in insulin secretion, by examining *SPARC* expression in relation to not just diabetes status but also glucose stimulated insulin secretion in primary human islets, and by assessing the effects of overexpression of *SPARC* on glucose stimulated insulin secretion (GSIS) in cultured rat beta cells.

## Materials and Methods

### Samples Used for Analysis

Human omental fat samples were obtained from subjects undergoing elective surgical procedures and collected via the Peninsula NIHR Clinical Research Facility human tissue bank. Samples for analyses of *SPARC* expression in liver and skeletal muscle were obtained as part of the tissue panel described below. Human islet samples were obtained from Procell Biotech (Newport Beach, USA). All islet samples were cultured overnight at source and scored for islet purity and viability. Islet preparations from this source were supplied with full clinical information, most importantly including glucose stimulated insulin secretion (GSIS) index measures following ELISA analysis. This is a measure of the ratio of insulin produced at 28 mM glucose compared with 2.8 mM glucose as described in Ihm *et al*, 2006 [26]. Characteristics of the islet donors are given in table 1.

**Table 1:** Characteristics of donors and samples for human primary islet collection.

Parameter	Non-diabetic subjects	Diabetic subjects
N	23	4
Sex (M/F)	10/13	3/1
Age (years)	39 (10.79)	52.75 (7.54)
Disease Duration (years)	N/A	2.665 (1.89)
BMI	30.2 (7.75)	39.6 (17.89)
Islet purity (%)	87	82
Islet viability (%)	91	93

M = Male, F = female, N = number of samples tested. BMI = Body Mass Index. HbA1c = Glycosylated haemoglobin.

### Ethics Statement

Ethical permission was granted from the South West Devon and Torbay Research Ethics Committee, for the human omental adipose samples. Written consent was obtained for all participants and agreed by the ethics board. All samples were treated, stored and processed according to the Declaration of Helsinki and Human Tissue Act regulations.

### RNA Preparation and Reverse Transcription

RNA was extracted from human omental adipose samples using the RNAeasy mini lipid extraction kit as recommend by the manufacturer (Qiagen, Crawley, UK). Liver and skeletal muscle samples were obtained directly as isolated RNA from AMS Biosciences (Abingdon, UK). RNA was extracted from primary human islets using the mirVana kit (Life Technologies, California, USA). All RNA samples were DNAse treated using the TURBO DNAse kit (Life Technologies, California, USA) prior to analysis, according to the manufacturer’s instructions. RNA samples were reverse transcribed using the Superscript III cDNA synthesis kit (Life Technologies, California, USA) in a volume of 20 µl, according to the manufacturer’s instructions.

### Design and Validation of a TaqMan Assay to Detect *SPARC* mRNA

A TaqMan assay was designed to the *SPARC* mRNA and purchased from the Assays-by-Design service available from Life technologies (California, USA). Probe and primer sequences are available on request. Assays were validated by 1 in 2 serial dilutions of pooled cDNA from multiple tissues to construct standard curves. Reactions were carried out on the ABI Prism 7900HT platform using SDS v2.3 software (Life Technologies, California, USA). Reactions contained 10 πl TaqMan Universal Mastermix (no AMPerase) (Applied Biosystems, Foster City, USA), 0.9 µM each primer, 0.25 µM probe and 2 µl cDNA reverse transcribed as above in a total volume of 20 µl. Cycling conditions were 50 cycles of 95°C for 10 mins, 15 seconds at 95°C and 1 min at 60°C using a 20 µl sample volume. The efficiency of the assay as determined from the curve gradient and the correlation between cDNA concentration and crossing point (r^2^ value) were assessed from the standard curves. The efficiency of amplification as given by the gradient of the standard curve was −3.7 and accuracy of quantification as given by the correlation co-efficient r^2^ was 0.99.

### Profiling of *SPARC* Expression in Human Tissues Relevant to Diabetes

SPARC expression was measured in pooled adipose tissue (n = 4), individual pancreatic islet samples (n = 8) and pooled liver (n = 1) and pooled muscle (n = 7). Expression profiles for *SPARC* were calculated by reference to the average crossing point of three replicates for each sample, relative to the mean crossing point of three separate endogenous control genes (*TBP*, *GUSB* and *B2M)*. Endogenous control genes were selected on the basis that their expression did not vary according to the parameters to be tested. Reaction conditions were as outlined above. Expression levels were calculated by the Comparative Ct method [27], and normalised to the levels of *SPARC* in adipose tissue.

### *SPARC* Expression According to Diabetes Status in Human Primary Islets

The expression level of *SPARC* mRNA was determined relative to 3 endogenous control genes in human primary islet samples from control individuals and from those with type 2 diabetes (participant characteristics are given in table 1). For this analysis, *SPARC* expression was calculated in individual islet samples. Endogenous control genes (*TBP*, *GUSB* and *B2M*), reaction and cycling condition were as described above. *SPARC* transcript levels were normalised to the median ΔCt for *SPARC* in the control samples. To account for the possibility that any expression differences could be due to potential reductions in beta cell mass in the islets from donors with diabetes [28], we also measured the expression of two genes with high expression in islets; phogrin (*PTPRN2*) and glucokinase (*GCK*). The association between diabetes status and the expression level of *SPARC* was assessed by Mann Whitney-U analysis. Non-parametric statistics were used to account for the low sample numbers.

### *SPARC* Expression and Basal Insulin or GSIS in Primary Human Islets from Individuals without Diabetes

*SPARC* expression in samples from control primary human islets (n = 23) was assessed by reference to the average crossing point of three replicates for each sample, relative to the mean crossing point of three separate endogenous control genes (*TBP*, *GUSB* and *B2M)*. Reaction conditions were as outlined above. *SPARC* expression levels were normalised to median ΔCt for *SPARC* across all the samples. Associations between *SPARC* expression and the increment in insulin secretion in response to a 28 mM glucose challenge as detailed in [26] were assessed by using linear regression on transformed data, adjusted for BMI. Since *SPARC* expression has previously been found to be positively regulated by insulin itself in adipose tissue [9], linear regression analysis adjusted for BMI was also carried out on the basal insulin measurement to assess the contribution of any regulatory effect of insulin in islets. A Bonferroni correction was carried out to control for multiple testing, since we were correlating SPARC expression with two outcome measures; insulin SI and basal insulin levels. A p-value of < = 0.025 was thus considered statistically significant.

### Overexpression of *SPARC* in Cultured Rat Beta Cells

Rat INS-1 beta cells were grown in RPMI-1640 medium containing 11 mM glucose, 10% foetal bovine serum, 2 mM L-glutamine, 100 U/ml penicillin and 100 µg/ml streptomycin. Cells were cultured at 37°C in 5% CO_2_ and were grown and maintained in 75-cm3 flasks. They were analysed or passaged when approximately 80% confluent.

Empty plasmid [pLNCX2] and plasmid containing the human *SPARC* construct were kindly provided by Dr David Allard, University of Exeter Medical School. To establish stable overexpression of *SPARC*, INS cells were seeded at 0.5×10^6^cells/well and allowed to attach for 24 hrs before transfection with 5 µg plasmid DNA in the presence of TransFast transfection reagent (Promega, Southampton, UK). Cells were grown in media supplemented with 0.15 mg/ml G418 (Invitrogen, Paisley, UK) to allow positive selection of transfected cells and creation of stable cell lines. Non-transfected INS cells cultured with G418 were used as a control for antibiotic efficiency. Two independent transfections were performed using the *SPARC* vector and empty vector respectively.

### Quantitative Real-time PCR Confirmation of *SPARC* Overexpression

Total RNA was extracted from *SPARC* transfected and empty vector transfected cells using the guanidinium-thiocyanate-phenol-chloroform extraction method [29]. Briefly, cells were homogenised by passing gently through a 21G needle in 1 ml Tri reagent (Life Technologies, California, USA) and RNA precipitated in line with manufacturer’s instructions. RNA quantity and purity was assessed spectrophotometrically using Nanodrop technology. Genomic contamination was removed from RNA using DNase I (Fermentas, Loughborough, UK) before 500 ng RNA was reverse transcribed using a Superscript VILO cDNA synthesis kit (Life Technologies, California, USA) in line with the manufacturer’s protocol.

cDNA was diluted 1∶2 in 0.01 M Tris HCl and all samples amplified in triplicate using expression assays specific for human (Hs00234160_m1) and rodent (Rn01470624_m1) *SPARC*. Gene expression levels were determined using the comparative Ct method [27], calibrating to *SPARC* expression within transfected cells (figure S1).

### Assessment of Insulin Secretion in Transfected Beta Cells

INS-1 cell lines expressing either pLNCX2 empty vector, or pLNCX2 h*SPARC*, were seeded in 24 well plates at 0.5×10^6^ cells/well and left to attach for 24 hrs. Cells were then pre-incubated with 0.5 ml of KREBs ringer buffer solution (125 mM NaCl, 4.74 mM KCl, 1 mM CaCl_2,_ 1.2 mM KH_2_PO_4_, 1.2 mM MgSO_4_, 5 mM NaHCO_3_, 25 mM Hepes, pH 7.4) containing 0.1% BSA and 2.8 mM glucose. Cell were then washed and acutely stimulated with 2.8 mM, 5.6 mM and 16.7 mM glucose or a combination of 100 mM IBMX (Sigma, Dorset, UK) and 2 mM forskolin (Sigma, Dorset, UK). After 1 hr media was sampled to measure insulin levels using a radioimmunoassay (individual reagents from Sigma, Dorset, UK), with radioactive insulin sourced from Millipore. The cells were washed in 0.5 ml of PBS, before the addition of 1 M NaOH, shaken at room temperature for 2 hrs, and the lysate used in a BCA assay (Pierce, Rockford, USA) to determine protein content. Insulin secretion levels were expressed as ng insulin/mg protein per well before normalising to insulin secretion at 2.8 mM glucose to give a stimulation index. Results were examined for statistical significance by 2-tailed t-test.

## Results

### *SPARC* mRNA is Present at Measurable Levels in Tissues Relevant to Diabetes

**Figure 1:**
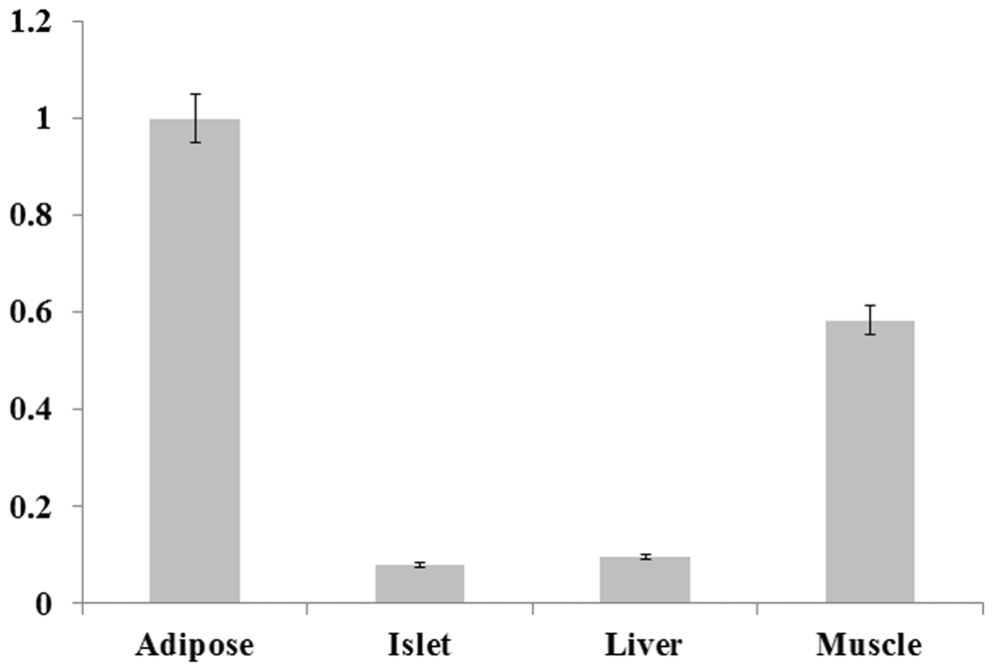
The expression profile of human *SPARC*. The expression patterns of *SPARC* mRNA in human adipose, pancreatic islet, liver and muscle tissues are given relative to the *TBP*, *GUSB* and *B2M* endogenous control genes. Expression level of *SPARC* in each tissue is expressed relative to *SPARC* levels in adipose tissue. Error bars are derived from the interquartile range of quantification.

*SPARC* mRNA was present at detectable levels in adipose tissue, pancreatic islets, skeletal muscle and liver (figure 1). Levels of *SPARC* were highest in adipose tissue and skeletal muscle, but were present at lower levels in the pancreatic islet and liver.

### *SPARC* Expression is Lower in the Primary Islets of People with Diabetes

*SPARC* was expressed at lower levels in primary islet samples from donors with diabetes compared with those from control donors. The mean expression levels of *SPARC* relative to the endogenous control genes was found to be 1.19 in control islets, compared with 0.43 in the islets from subjects with diabetes; p = 0.049. We found no differences in the expression levels of either Phogrin or Glucokinase between islet samples from subjects with and without diabetes (p = 0.83 and p = 0.53 respectively) which suggests no significant reduction in islet cells in subjects with diabetes. Circulating SPARC is known to increase in obesity on the basis of increased expression and secretion of *SPARC* in adipose tissue [9]. Whilst donors with diabetes had a higher BMI than control donors, no significant increase of *SPARC* expression due to increased BMI was noted in our samples.

### *SPARC* Expression is Correlated with Glucose-stimulated Insulin Secretion in Primary Islets

**Figure 2:**
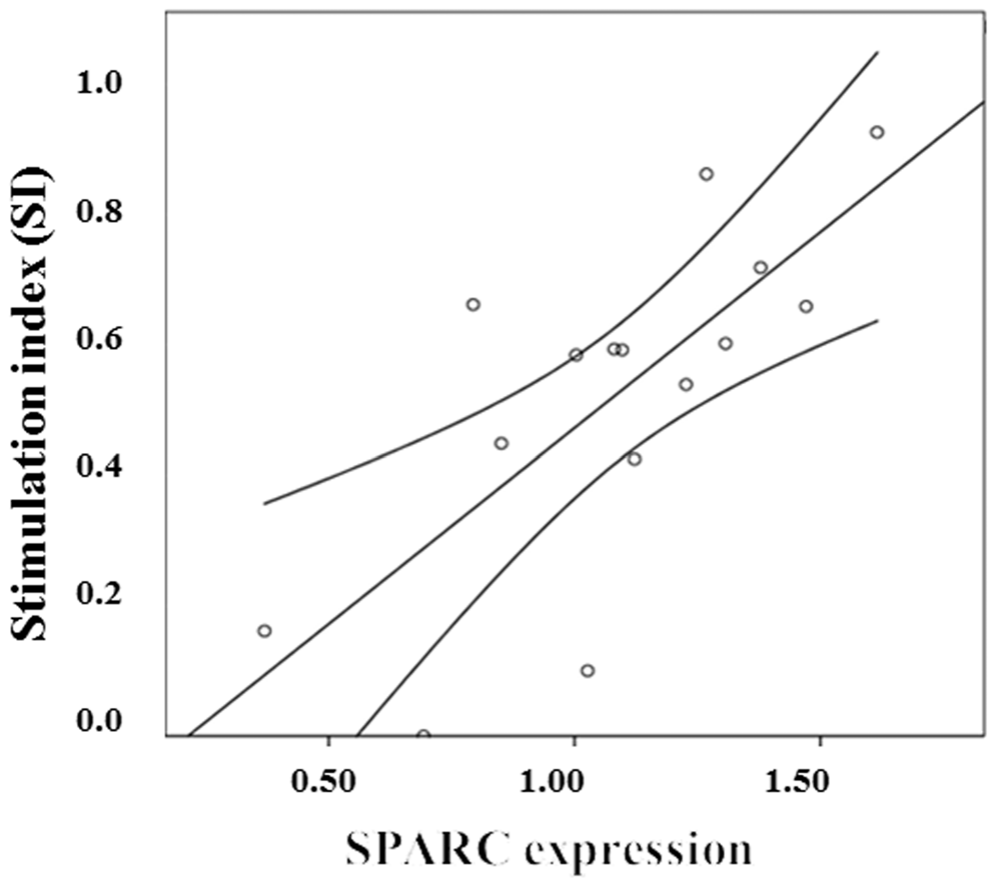
The association of *SPARC* expression on glucose stimulated insulin secretion in primary human islets. The figures on the Y axis refer to insulin stimulation index (SI) following a 28 mM glucose challenge. The expression level of *SPARC* is given on the X axis. Expression levels were calculated relative to the endogenous controls *GUSB, B2M* and *PP1A* and normalised to the mean level of *SPARC* across the cohort.

*SPARC* expression was positively correlated with glucose stimulated insulin secretion (GSIS) index measures as determined by ELISA analysis (table 2; figure 2). The beta coefficient following linear regression analysis adjusted for BMI was 0.68; p = 0.007. In contrast to the situation in adipose tissue, we found no correlation of *SPARC* expression with basal insulin levels pre-glucose challenge; beta coefficient −0.43, p = 0.153 (table 2).

**Table 2:** Glucose stimulated insulin secretion and basal insulin according to *SPARC* expression in primary human islet cultures.

Transcript	Effect size (beta co-efficient)	Standard Error	p-value	Correlation co-efficient (r^2^)
**Glucose stimulated insulin increment**				
*SPARC*	0.68	0.165	***0.007***	0.54
**Basal insulin**				
*SPARC*	−0.43	0.227	0.153	0.24

Associations between Glucose-stimulated insulin secretion and basal insulin levels were assessed by linear regression analysis, adjusted for BMI. Significant associations are indicated in bold italic type.

### Overexpression of Human *SPARC* in INS-1 Cells Increases Glucose-stimulated Insulin Secretion

**Figure 3:**
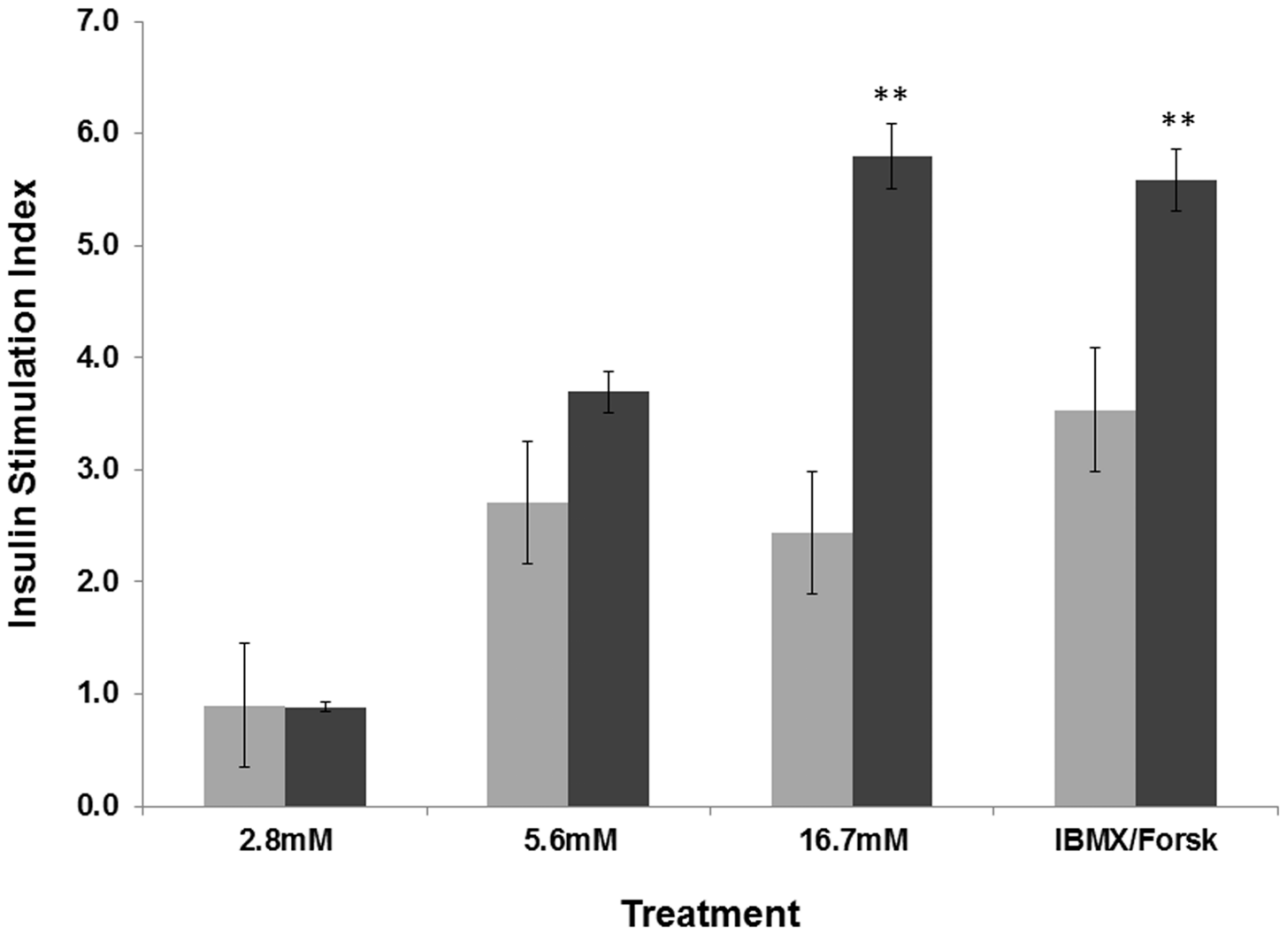
Changes in the insulin stimulation index in *SPARC*-transfected rat beta cells under different glucose conditions. The figure shows the stimulation index (SI) of transfected rat beta cells (INS-1) in response to 2.8 mM, 5.6 mM and 16.7 mM glucose or IBMX/forskolin. Results from cells transfected with empty vector are given in light grey, and those from *SPARC*-transfected cells are given in dark grey. Error bars represent standard error of measurements. Data are combined results from the two transfected cell lines. Statistical significance is indicated by stars.

The rat pancreatic β-cell line, INS-1 was used to directly determine the influence of *SPARC* on insulin secretion. Two independent stably transfected INS-1 cell lines pLNCX2 SPARC#1 and pLNCX2 SPARC#2, overexpressing human *SPARC* were created (see figure S1). The *SPARC* overexpressing cells showed an 2.4-fold increase in insulin secretion stimulated by 16.7 mM glucose (Stimulation Index 5.8 compared with 2.4 in vector-only cells; p = 0.002), but not 2.8 mM glucose or 5.6 mM glucose compared to empty vector control cells (figure 3), suggesting that its effects are specific to high glucose concentrations. *SPARC* overexpressing cells also demonstrated an 1.6-fold increase in insulin secretion in response to stimulation by IBMX/forskolin (Stimulation Index 5.6 compared with 3.5; p = 0.004; figure 3).

## Discussion

In this study we report a potential novel role for the matricellular protein SPARC in moderation of insulin secretion in pancreatic islets. The *SPARC* gene is expressed at detectable levels in primary human islets and was found to be lower in the islets of patients with type 2 diabetes, despite a higher BMI in these subjects. *SPARC* expression was also positively correlated with glucose-stimulated insulin secretion (GSIS), in human primary islet cultures. Furthermore, overexpression of *SPARC*, in cultured rat beta cells (INS-1) resulted in a 2.4-fold increase in glucose stimulated insulin secretion compared to control cells. Insulin secretion stimulated by IBMX/forskolin in INS-1 cells was also potentiated by SPARC overexpression.

SPARC is known to be induced by insulin in human adipose tissue [9]. Interestingly, in our study, we found no evidence that *SPARC* expression level was correlated with basal insulin levels in primary human islets (table 2). In adipose tissue, insulin levels are positively correlated with SPARC levels in adipose tissue [9], whereas we have found that basal insulin is negatively correlated with *SPARC* expression in human islets, indicating some degree of tissue specificity. This is entirely consistent with a known feature of SPARC function, in that it can play apparently opposing roles depending on the cell type and intercellular environment [30]. Whilst the expression levels of *SPARC* were lower in islets from donors with diabetes, this may not necessarily impact on the function of SPARC in the islet or could indicate a consequence of altered splicing induced by a disturbance in glucose or lipid homeostasis, which will need to be further evaluated in subsequent studies.

SPARC could be playing a part in moderation of beta-cell function by several potential mechanisms. Our observation that insulin secretion is increased in *SPARC*-transfected rat beta cells in response to IBMX/Forskolin may suggest that the mode of action may involve alternative regulatory pathways such as the amplification pathway [31], although this suggestion would require more experimental evidence to prove definitively, and will form the basis for future studies. The amplification pathway acts by increasing the effect of high cellular calcium on insulin vesicle exocytosis once glucose has reached a threshold value. The amplification pathway is known to be impaired in beta-cells of animal models of type 2 diabetes and there is also some evidence that it may also be affected in the islets of patients with type 2 diabetes [31]. This is concordant with our own observations that *SPARC* expression is reduced in the islets of patients with type 2 diabetes.

SPARC is also known to have roles as a survival factor. Studies in fruit flies have demonstrated that cells can secrete SPARC in response to apoptotic stimuli, which protects them from cell death [32]. This has been confirmed in human studies in breast cancer, where SPARC has been shown to cause the AKT-mediated phosphorylation of the *MDM2* gene product, and subsequently the negative regulation of the tumour suppressor p53 [22]. Apoptosis is thought to be one of the major factors in decline of beta cell mass in both type 1 and type 2 diabetes [33] and the failure of beta cell mass to expand in order to compensate for increasing insulin resistance is another key factor in the development of type 2 diabetes [34]. SPARC has also been shown to have proliferative, as well as anti-apoptotic effects; depletion of SPARC by siRNA technology has been demonstrated to cause cell cycle arrest by activation of p53 and the p21Cip1/Waf1 pathway in melanoma cells [35].

SPARC could also be affecting beta-cell function by affecting the differentiation state of pancreatic beta cells. Beta cell development in embryogenesis, and also neogenesis of beta cells as a response to insulin resistance is known to be influenced by the activity of several metalloproteinases [20], [21]. This is an important group of proteins that are involved in the degradation and remodelling of the extracellular matrix and have previously been demonstrated to be regulated by SPARC [19]. Again, dedifferentiation of pancreatic beta cells to more embryonic forms has been described to be a feature of type 2 diabetes in both mouse and man [24]. In addition to its role in the regulation of *MMP* genes, SPARC has also been shown to regulate the activity of the NOTCH/STAT3 pathway [36]. This is relevant because of the recent data implicating elevated Notch and related signalling pathways in the dedifferentiation of beta cells in type 2 diabetes [37].

Finally, SPARC also has a role in WNT signalling. SPARC has been shown to enhance the accumulation and nuclear translocation of beta-catenin resulting in the activation of its partner genes such as *TCF4* (*TCF7L2*) [13]. This is noteworthy since polymorphisms in the *TCF7L2* gene have been demonstrated in genome wide association studies (GWAS) to show the largest association with susceptibility to type 2 diabetes in several populations [38] and have also been demonstrated to influence insulin exocytosis in human and rodent islets and in cell lines [39], [40]. Interestingly, several other genes in pathways regulated by SPARC (cell cycle/survival; *TP53*, metalloproteinase activity; *ADAMTS9* and beta-cell differentiation/neogenesis; *NOTCH2*) are represented in the GWAS for type 2 diabetes [38].

The strength of this study is the identification of a potential novel role for SPARC in the regulation of insulin secretion relevant for the onset of Type 2 diabetes. This may oppose the role of SPARC in adipose tissue where it is thought to increase fibrosis by increased deposition of insoluble collagen fibers, in contrast islets of patients with diabetes are not characterised by collagen deposition but an accumulation of amyloid (Clark A, 2001). The adipose tissue fibrosis furthers ectopic deposition of triglycerides in inappropriate tissues [10], [16]. Whilst obesity related adipose tissue fibrosis may well attribute to insulin resistance as also shown by increased levels of ECM components such as collagen VIalpha3 [41], the multifunctional protein SPARC appears to influence more than one pathway of glucose and insulin metabolism. In line with this, a reduction in SPARC activity has also been associated with altered glucose metabolism. *SPARC* knockout mice demonstrate retarded glucose uptake following feeding and an impaired intraperitoneal glucose tolerance test [42]. In addition, recent studies have found a role for SPARC in the regulation of GLUT4-mediated glucose transport, by modification of AICAR-mediated phosphorylation of AMPK, where the relationship between SPARC and AMKP is controlled by an autoregulatory loop, as determined by siRNA studies [43].

Our results do not suggest an increase of *SPARC* expression in islets of subjects with Type 2 diabetes as may have been expected from its antiproliferative and profibrotic action in adipose tissue [9]. After reducing the influence of obesity by BMI matching and/or statistical adjustments for BMI in interpretation of our data and study of expression in human islets expressions rather than adipocytes, we found a significant reduction of *SPARC* expression in islets from subjects with diabetes. This does not reflect the pattern of adipose tissue expression of *SPARC,* which is positively associated with HOMA-IR [9], or the serum levels of SPARC, which are positively correlated with HbA1C in men without diabetes [17] and increased in subjects with newly diagnosed diabetes [11] and serum protein levels appear to differ from the RNA expression in peripheral blood lymphocytes. Since adipose tissue levels of SPARC are up-regulated by high insulin levels (causing increased secretion of *SPARC* from adipose tissue to the serum), the inverse correlation between basal insulin levels and *SPARC* expression we note in control islets, may indicate that the relationship between *SPARC* expression and insulin levels is different in pancreatic islets.

To conclude, we report that the SPARC gene is expressed at detectable levels in human primary islets, and is associated with diabetes status and glucose stimulated insulin secretion in this tissue. Furthermore, overexpression of *SPARC* in cultured rat beta cells caused a 2.4-fold increase in insulin secretion in high glucose conditions, which was also mirrored when the SPARC-transfected beta cells were treated with IBMX/Forskolin. We suggest that these data point towards a previously uncharacterised potential role for SPARC in moderation of insulin secretion, separate from the anti-proliferative and profibrotic function of this protein in adipose tissue where it appears to be a moderator of insulin resistance.

## Supporting Information

Figure S1 **Expression of human** ***SPARC*** **mRNA in transfected beta cells.** The levels of human *SPARC* mRNA in INS-1 cells induced by transfection of human *SPARC* sequences compared to the transfection of empty vector is given on the Y axis, and the nature of the transfection (i.e. *SPARC* constructs or empty vector) are given on the X-axis. Expression levels in each transfected sample were normalised to the levels of *SPARC* expression within *SPARC* transfected cells.
(PPTX)
