# Supplementary figures and images for: A Role for SPARC in the Moderation of Human Insulin Secretion
Source: PLoS One. 2013 Jun 28;8(6):e68253. doi: 10.1371/journal.pone.0068253 (PMC3695891; doi:10.1371/journal.pone.0068253)

## Slide 1
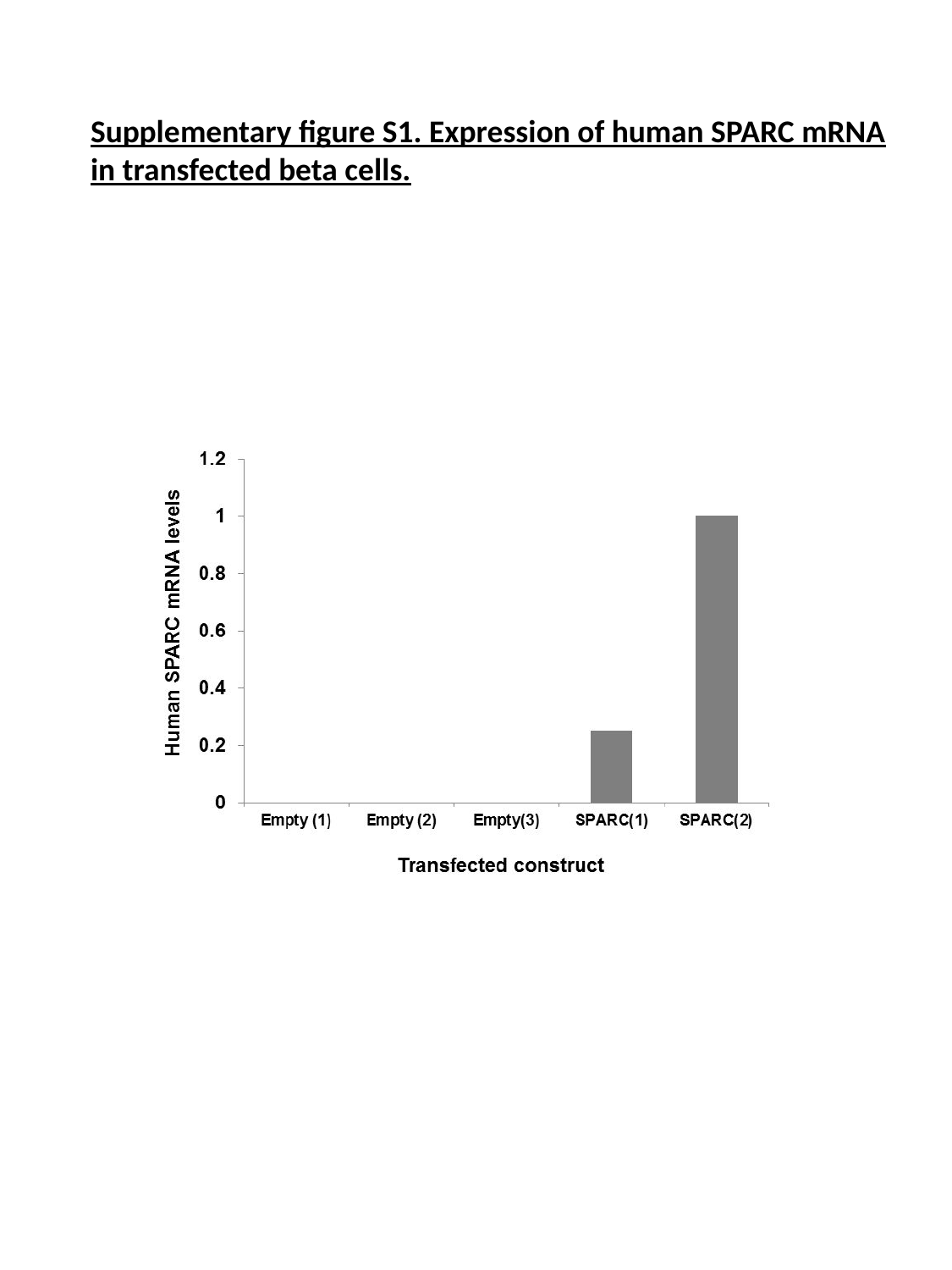

Supplementary figure S1. Expression of human SPARC mRNA in transfected beta cells.

Supplement: Figure S1 — Expression of human SPARC mRNA in transfected beta cells. The levels of human SPARC mRNA in INS-1 cells induced by transfection of human SPARC sequences compared to the transfection of empty vector is given on the Y axis, and the nature of the transfection (i.e. SPARC constructs or empty vector) are given on the X-axis. Expression levels in each transfected sample were normalised to the levels of SPARC expression within SPARC transfected cells. (PPTX) [file pone.0068253.s001.pptx]
